# Cancer Associated Aberrant Protein O-Glycosylation Can Modify Antigen Processing and Immune Response

**DOI:** 10.1371/journal.pone.0050139

**Published:** 2012-11-26

**Authors:** Caroline B. Madsen, Cecilie Petersen, Kirstine Lavrsen, Mikkel Harndahl, Søren Buus, Henrik Clausen, Anders E. Pedersen, Hans H. Wandall

**Affiliations:** 1 Department of Cellular and Molecular Medicine, Faculty of Health Sciences, University of Copenhagen, Copenhagen, Denmark; 2 Department of International Health, Immunology and Microbiology, Faculty of Health Sciences, University of Copenhagen, Copenhagen, Denmark; UCLA, United States of America

## Abstract

Aberrant glycosylation of mucins and other extracellular proteins is an important event in carcinogenesis and the resulting cancer associated glycans have been suggested as targets in cancer immunotherapy. We assessed the role of O-linked GalNAc glycosylation on antigen uptake, processing, and presentation on MHC class I and II molecules. The effect of GalNAc O-glycosylation was monitored with a model system based on ovalbumin (OVA)-MUC1 fusion peptides (+/− glycosylation) loaded onto dendritic cells co-cultured with IL-2 secreting OVA peptide-specific T cell hybridomas. To evaluate the *in vivo* response to a cancer related tumor antigen, Balb/c or B6.Cg(CB)-Tg(HLA-A/H2-D)2Enge/J (HLA-A2 transgenic) mice were immunized with a non-glycosylated or GalNAc-glycosylated MUC1 derived peptide followed by comparison of T cell proliferation, IFN-γ release, and antibody induction. GalNAc-glycosylation promoted presentation of OVA-MUC1 fusion peptides by MHC class II molecules and the MUC1 antigen elicited specific Ab production and T cell proliferation in both Balb/c and HLA-A2 transgenic mice. In contrast, GalNAc-glycosylation inhibited the presentation of OVA-MUC1 fusion peptides by MHC class I and abolished MUC1 specific CD8+ T cell responses in HLA-A2 transgenic mice. GalNAc glycosylation of MUC1 antigen therefore facilitates uptake, MHC class II presentation, and antibody response but might block the antigen presentation to CD8+ T cells.

## Introduction

Cancer vaccines hold great promise for long lasting therapeutic efficacy [1]. Current experimental cancer vaccines primarily aim to elicit cellular immunity through induction of specific CD8+ T cells [2]–[5]. However, increasing evidence demonstrates the value of antibodies in tumor eradication [6], [7]. Consequently, there is a growing interest in designing vaccines that can activate both CD4+ and CD8+ T cells and thereby simultaneously elicit humoral and cellular cancer immunity [8]. Altered proteins presented on cancer cells are important tumor antigens, which can be targeted by vaccines and therapeutic antibodies. Most cell surface proteins are glycosylated, and malignant transformation of cells is always accompanied by alterations of posttranslational modifications of proteins [9]. Aberrant mucin-type O-glycosylation represents one of the most abundant posttranslational cancer associated changes creating a diverse set of molecular structures found on the surface of cancer cells, but not on normal cells [9], [10]. As a result, the specific pattern of the cancer associated short glycan structures on cancer-associated proteins produces novel glycopeptide epitopes that can be targeted by the immune system [11], [12].

Cancer associated glycans affect cancer immunity in several ways. The aberrant glycans are immunogenic by themselves, and truncated O-glycans (Tn: GalNAc-S/T; STn: NeuAcα2,6GalNAc-S/T; and T: Galβ1,3 GalNAc-S/T) are recognized as pan-carcinoma antigens to which circulating antibodies are found at elevated levels in cancer patients. Aberrant glycans may also induce novel epitopes on proteins by causing conformational change or by the creation of new O-glycopeptide epitopes [13], [14]. In addition, cancer associated glycans can be included in the design of immunogenic epitopes that can induce *in vivo* anti-tumor immune responses [15]. An important basis for glycan induced immunity is that aberrant glycans may bind lectin receptors. For example, macrophage galactose binding lectin (MGL)-mediates uptake of specific O-glycoproteins in dendritic cells [10], [16]. Indeed, dendritic cells are the most potent antigen presenting cells for priming of naïve CD8+ and CD4+ T cells. They carry a range of carbohydrate receptors of the C-type lectin family (reviewed in [17]) and possess a unique ability to cross-present extracellular antigens to CD8+ T cells [18]. The selective uptake of GalNAc-glycosylated antigens (Tn-antigens) by the common human and murine dendritic cell (DC) C-type lectin MGL, [19], [20] makes it possible to induce cancer specific glycopeptide antibodies in mice and man through induction of a CD4+ Th cells [11], [13], [14], [21]. Such Tn-glycopeptide specific antibodies mediate antibody dependent cytotoxicity [11], [12], [22], [23], and the occurrence of natural glycopeptide specific antibodies in cancer patients is suggested to be associated with increased survival [21]. In contrast, very few CD8+ T cells targeting the glycopeptide epitopes of extracellular tumor antigens (e.g. mucin 1 (MUC1)) have been described [24], [25].

The cancer associated glycoprotein MUC1 is an important model molecule in cancer immunotherapy and is aberrantly glycosylated in most adenocarcinomas [26]. The extracellular domain of MUC1 consists of 20–120 tandem repeats (VNTR), each containing 20 amino acids with 5 potential O-glycosylation sites [27]. Immunization of B6.Cg(CB)-Tg(HLA-A/H2-D)2Enge/J (HLA-A2 transgenic mice) [28] and chimpanzees [29] with non-glycosylated MUC1 antigens has elicited MUC1 peptide specific CD4+ and CD8+ T cell responses and several CD8+ T cell epitopes have been identified within the extracellular and intracellular domains of MUC1 [28], [30]–[33]. In MUC1 transgenic mice the response to non-glycosylated MUC1 peptide based vaccines has, however, been very low with small or undetectable CD4+ and CD8+ T cell responses [34], [35]. Non-glycosylated MUC1 peptide conjugated to the immunogenic glycoconjugate mannan elicited strong immune responses including CD8+ T cells [31]. In humans, the non-glycosylated MUC1 peptide vaccines have primarily evoked humoral and CD4+ T cell responses [36]–[38], while viral and DC based vaccines have also generated cytotoxic T lymphocyte (CTL) responses [39]–[44]. The induction of CD8+ T cells was only observed in few patients or after several rounds of re-stimulation *in vitro* [39]–[44], however, several of the vaccines have demonstrated efficacy, albeit limited [38]–[41], [44]–[57]. Two viral vaccines expressing MUC1, either in combination with IL-2 (TG4010) [41], [48]–[51] or in combination with carcinoembryonic antigen (PANVAC) [52], have demonstrated clinical effect in patients with non small-cell lung and pancreatic cancer [39]–[41], [48]–[52]. In addition, clinical effect has also been demonstrated with the liposomal vaccine (BLP25) consisting of a 25 mer non-glycosylated peptide from the MUC1 tandem repeat region [53]–[57]. Interestingly, in a small clinical study in early stage breast cancer patients immunized with mannan-MUC1, the vaccine induced both humoral and T-cell responses to non-glycosylated MUC1 and eliminated recurrence of disease [45].

Most previous vaccines have targeted non-glycosylated MUC1, although this might not be the most cancer specific target. Targeting cancer associated glycopeptide epitopes in MUC1 such as GalNAc-MUC1 [11], [12], [58] would be favorable, but biosynthesis of MUC1 glycopeptide epitopes expressed by tumors has been hampered by lack of relevant technologies and high costs. Thus, it is currently not clear how glycans will affect the uptake, presentation, recognition, and induction of immune responses to CD8+ T cell epitopes. Although the presence of glycans might have a positive effect on the uptake of glycosylated antigens [19], [20], [59], glycans could compromise entry into the immuno-proteasome [60], proteasomal cleavage, TAP-mediated translocation, and peptide loading onto MHC class I molecules [60], [61].

In this study we have examined the consequence of MUC1 GalNAc-glycosylation on known CD8+ and CD4+ T cell activating antigens. We took advantage of the ovalbumin model system using OVA-MUC1 fusion peptides containing the immunodominant MHC class I (SIINFEKL) and MHC class II (ISQAVHAAHAEINEAGR) OVA epitopes as readout for antigen uptake and processing. The epitopes were flanked by GalNAc glycosylated or non-glycosylated MUC1 derived peptide sequences. In addition, we examined the effect of GalNAc glycosylation of a physiological tumor associated MUC1 glycopeptide known to induce CD4+ and CD8+ T cell responses. This peptide (AHGVTSAPDNRPALGSTAPPVHNV) has close sequence homology with the tandem repeat of MUC1, but contains an HLA-A2 epitope lacking in the tandem repeat. We present data suggesting that GalNAc residues aid antigen uptake and MHC class II presentation for the generation of a potent cancer-specific antibody response, and block presentation to CD8+ T cells.

## Results

### GalNAc-glycosylation Improves CD4+ T cell Responses

**Figure 1:**
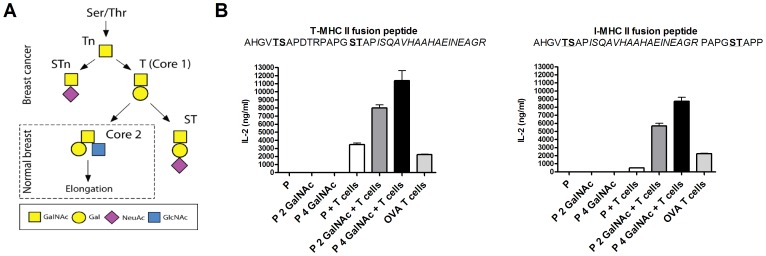
Peptide glycosylation enhances the activation of antigen specific CD4+ T cell hybridoma. A) O-glycan synthesis overview. B) IL-2 production from OVA specific CD4+ T cell hybridoma (BO.97.10) co-cultured with bone marrow derived DCs pulsed with two peptide variants with and without 2 and 4 glycan residues (GalNAc). Full length OVA used as a positive control. Individually cultured T cells and DCs had an OD value equal to the background.

We first investigated the role of GalNAc-glycosylation on peptide antigen processing and peptide presentation on MHC class II molecules (Figure 1). The potent I-A^b^ binding OVA peptide was fused to a MUC1 derived sequence with and without GalNAc-glycosylation (Table 1) and was loaded on DCs which were co-cultured with ovalbumin peptide/MHC class II complex specific T cell hybridomas. DCs loaded with glycopeptides increased the OVA specific CD4+ T cell hybridoma response to the OVA derived peptide when compared with non-glycosylated peptide, regardless of where the OVA epitope was located in the peptide sequence (Figure 1B). Peptides containing four GalNAc-residues induced a higher response than peptides containing two GalNAc residues [62], [63] (Figure 1B). Even very low concentrations (30 nM) of glycopeptides stimulated the CD4+ T cell hybridoma, while low concentrations of the non-glycosylated peptide induced no response (data not shown).

**Table 1:** Peptide sequences for DC T cell hybridoma co-culture.

Name	Peptide	2 GalNAc	4 GalNAc
1) I-SIINFEKL-MUC1	AHGVTSAPSIINFEKLPAPGSTAPP	AHGVTSAPSIINFEKLPAPGSTAPP	AHGVTSAPSIINFEKLPAPGSTAPP
2) T-SIINFEKL-MUC1	AHGVTSAPDTRPAPGSTAPSIINFEKL	AHGVTSAPDTRPAPGSTAPSIINFEKL	AHGVTSAPDTRPAPGSTAPSIINFEKL
3) I-MHC II fusion	AHGVTSAPISQAVHAAHAEINEAGRPAPGSTAPP	AHGVTSAPISQAVHAAHAEINEAGRPAPGSTAPP	AHGVTSAPISQAVHAAHAEINEAGRPAPGSTAPP
4) T-MHC II fusion	AHGVTSAPDTRPAPGSTAPISQAVHAAHAEINEAGR	AHGVTSAPDTRPAPGSTAPISQAVHAAHAEINEAGR	AHGVTSAPDTRPAPGSTAPISQAVHAAHAEINEAGR

1 and 2 (H2-Kb restricted fusion), 3 and 4 (I-Ab restricted fusion). Underlining indicate sites of glycosylation. Internal (I), terminal (T), N-Acetylgalactosamine (GalNAc).

### GalNAc Glycosylation Inhibits the Processing and Presentation of a MHC Class I Binding Peptide *in vitro*

**Figure 2:**
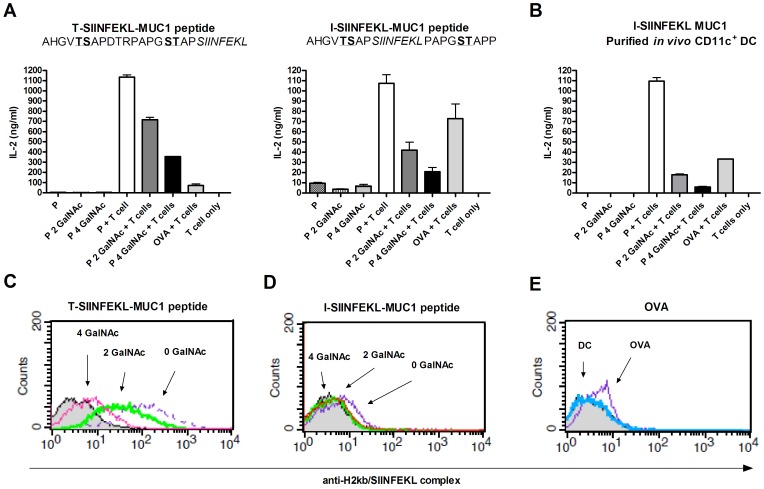
Peptide glycosylation inhibits activation of antigen specific CD8+ T cell hybridoma. IL-2 production from OVA specific CD8+ T cell hybridoma (RF 33.70) co-cultured with bone marrow derived DCs (A) or CD11c+ DCs purified from mouse spleen (B). DCs were pulsed with two peptide variants with and without 2 and 4 glycan residues (GalNAc). Full length OVA was used as a positive control. Individually cultured T cells and DCs had an OD value equal to the background. C, D) Surface expression by flow cytometry of SIINFEKL in the H2kb peptide binding groove on DCs after pulsing with the two peptide variants (non-glycosylated peptide (thin purple dashed line), peptide with 2 GalNAcs (thick green line), or 4 GalNAcs (pink line)). E) Surface expression of SIINFEKL in the H2kb peptide binding groove on DCs without pulsing (blue line) and after pulsing with OVA control (purple line). Gray histograms represent cells stained only with secondary antibody.

The same model system was used to investigate the influence of GalNAc glycosylation on MHC class I presentation. *In vitro* generated, as well as purified CD11c+ spleen DCs, were loaded with GalNAc glycosylated or non-glycosylated MUC1-SIINFEKL fusion peptides (Table 1). In contrast to the results obtained with MHC class II presentation, GalNAc glycosylation of MUC1-SIINFEKL resulted in lower activation of SIINFEKL/H2-K^b^ specific T cell hybridomas when compared to non-glycosylated peptides regardless of the origin of the DCs (Figure 2A–B). We next tested how the location of glycan residues relative to the MHC class I binding epitope affected the CD8+ T cell response. DCs were stimulated using fusion peptides with SIINFEKL either flanked by glycosylation sites or localized to the C-terminal end of the peptide. Fusion peptides with terminal SIINFEKL demonstrated an increased T cell hybridoma response compared to fusion peptide with SIINFEKL localized inside the MUC1 repeat regardless of whether the peptides were glycosylated or not. Flow cytometric analysis of the SIINFEKL/H2-K^b^ complex on DCs after peptide processing confirmed lower MHC class I presentation of GalNAc-glycosylated peptides compared with non-glycosylated peptides (Figure 2C–E).

### GalNAc Glycosylation of a MUC Derived Antigen Elicits Specific IgG Antibodies and T cell Proliferation *in vivo*

**Figure 3:**
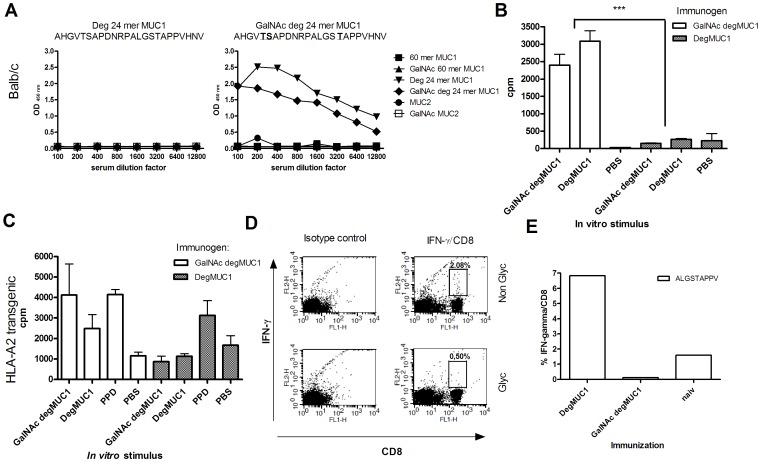
*In vivo* response to degMUC1 in WT Balb/c and HLA-A2 transgenic mice. A) Serum reactivity to different mucin glycoforms from Balb/c mice immunized with degMUC1+/− GalNAc by ELISA. Data are representative of a minimum of 4 Balb/c mice immunized with each antigen. (B) T cell proliferation from mice Balb/c mice or (C) HLA-A2 mice immunized with GalNAc degMUC1 (white bars) and non-glycosylated degMUC1 (grey bars) for each peptide used for *in vitro* re-stimulation (100 ug/ml) as indicated on the x-axis. D) Lymphocytes from immunized HLA-A2 mice pulsed with the 9 mer degMUC1 peptide, ALGSTAPPV, with and without glycosylation. DegMUC1 specific CD8+ T cells were selected based on anti-CD8 Ab (x-axis) and anti-IFNγ Ab (y-axis) after 4 hrs of Golgi stop treatment. E) Spleen cells after 24 days of re-stimulation with the non-glycosylated ALGSTAPPV peptide. All T cell data is generated from spleen or lymph nodes from at least 4 mice, but in most cases 6 mice.

The amino acid sequence of the 24 mer synthetic MUC1 peptide (degMUC1) resembles the MUC1 tandem repeat, however, its amino acid sequence is slightly altered resulting in an HLA-A2 epitope (ALGSTAPPV) [28]. DegMUC1 is therefore optimal for inclusion in human cancer vaccines aiming at inducing MUC1 specific CTL responses. To investigate the role of GalNAc glycosylation *in vivo* for this MUC1 model tumor antigen, we immunized Balb/c mice and HLA-A2 transgenic mice with degMUC1 with and without GalNAc glycosylation. Balb/c mice immunized with GalNAc-glycosylated degMUC1 developed a strong IgG response specific for degMUC1, while there was no response in mice immunized with the non-glycosylated degMUC1 (Figure 3A). Immunizations with KLH-conjugated glycosylated and non-glycosylated degMUC1 resulted in an IgG response in both mouse strains, though the glycosylated variant resulted in a more prominent response (data not shown). In agreement with the humoral IgG response, Balb/c mice immunized with GalNAc degMUC1 elicited significant T cell proliferation after re-stimulation with either GalNAc or non-glycosylated degMUC1 (P≤0,0004). No T cell proliferation was detected in mice immunized with non-glycosylated degMUC1 (Figure 3B). Similar, a higher proliferative response was seen in HLA-A2 transgenic mice immunized with glycosylated compared to non-glycosylated degMUC1 verifying the positive effect of GalNAc glycosylation on the CD4+ T cell reactivity and humoral immune response (Figure 3C). As a control tuberculin purified protein derivative (PPD) stimulation resulted in similar T cell proliferation in both groups of HLA-A2 transgenic mice.

### GalNAc Glycosylation of a MUC1 Derived Antigen Block the CD8+ T cell Response *in vivo*

We next examined the CD8+ T cell response after immunization of HLA-A2 transgenic mice with degMUC1 (+/−GalNAc glycosylation) followed by re-stimulation with the degMUC1 9 mer CD8+ epitope ALGSTAPPV (+/− GalNAc glycosylation). Only the mice that were immunized and re-stimulated with the non-glycosylated degMUC1 peptide generated IFN-γ producing, ALGSTAPPV specific, CD8+ T cells, while no response was seen in mice immunized with the GalNAc glycosylated degMUC1 (Figure 3D). T cells from immunized mice were expanded by re-stimulation *in vitro* for a total of 24 days with ALGSTAPPV confirming that only the mice immunized with the non-glycosylated degMUC1 responded to re-stimulation with ALGSTAPPV (Figure 3E). Next, we analyzed stability and affinity of the HLA-A*02∶01-ALGSTAPPV complex with and without GalNAc glycosylation. The HLA-A*02∶01-ALGSTAPPV complex had a half-life of 19 hrs and an affinity of 100 nM, whereas the HLA-A*0201-ALGST[GalNAc]APPV complex had a half-life of 11 hrs with an affinity of 300 nM. Thus, ALGST[GalNAc]APPV is still a solid intermediate binder and should be able to elicit a CD8+ T cell response based on MHC binding data.

### High Density GalNAc Glycosylation Increases Uptake of MUC1 and MUC2 Derived Peptides but Inhibits MHC Class I Presentation

**Figure 4:**
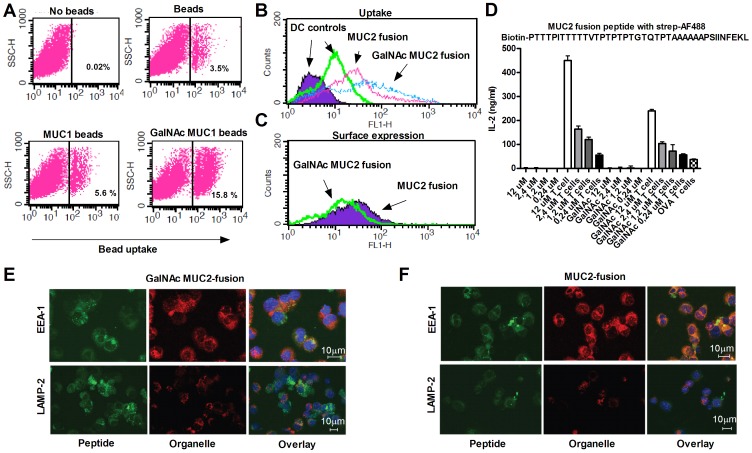
Increased density of peptide glycosylation increases uptake but inhibits activation of antigen specific CD8+ T cell hybridoma. A) DC uptake of uncoated fluorescent beads, MUC1-fluorescent beads, or GalNAc MUC1 fluorescent beads. DCs without beads were used as reference. B) The uptake of MUC2 fusion peptide +/−GalNAc was evaluated by flow cytometry after 48 hrs of peptide pulsing. Unstained DCs (purple filled) and DCs with no peptide load (green) were used as a background control. C) Evaluation of the surface expression by flow cytometry of SIINFEKL in the H2kb peptide binding groove. DCs were pulsed with the MUC2 fusion peptide (highest concentration from D) with GalNAc (green line) and without GalNAc (purple filled) 48 hrs before the staining. D) IL-2 production from SIINFEKL specific CD8+ T cell hybridoma (RF 33.70) co-cultured with bone marrow derived DCs pulsed *in vitro* for two days with MUC2 fusion peptide both with and without glycosylation in a concentration gradient. Full length OVA was used as a positive control. Representative data of at least two independent experiments is shown. E, F) Confocal imaging of DC internalized GalNAc MUC2 fusion (E, green) and MUC2 fusion (F, green) co-stained with endosomal marker EEA-1 (red) and lysosomal marker LAMP-2 (red).

Density of glycosylation may affect uptake mediated by lectin receptors, such as MGL. Therefore, we speculated that the lack of CD8+ T cell responses against GalNAc degMUC1 peptides could be caused by low uptake of the short GalNAc modified MUC1 peptides. Although, a limited number of GalNAc residues are enough to induce a potent I-Ab response, it might be insufficient for the cross presentation required to induce an H-2K^b^ response. To test the effect of GalNAc density we pulsed DCs with GalNAc-glycosylated biotinylated 60 merMUC1-peptide covering three MUC1 tandem repeats (60 merMUC1). While GalNAc incorporation did not increase uptake of the monomer form of 60 merMUC1, increased uptake was seen when complex formation was induced by streptavidin (data not shown). Next, we tested uptake of fluorescent beads coated with MUC1 with and without glycosylation. Beads coated with GalNAc MUC1, which provide a very high density of GalNAc MUC1, resulted in a marked increase in uptake compared to non-glycosylated MUC1 (Figure 4A). The effect of multiple GalNAc residues on DC uptake prompted us to test whether synthetic peptides containing multiple GalNAc residues could overcome the lack of cross presentation of CD8+ T cell epitopes observed with the shorter MUC1 peptides. A MUC2 fusion peptide containing multiple GalNAc glycosylations sites was designed and *in vitro* glycosylated to produce a peptide with and without 9–10 GalNAc residues localized in the MUC2 sequence. The GalNAc MUC2 fusion peptide was readily taken up by the DCs (Figure 4B). However, GalNAc glycosylation still inhibited surface expression of SIINFEKL/H2-K^b^ complexes and T cell hybridoma activation was lower (Figure 4C–D). Imaging of internalized MUC2 fusion peptide with and without GalNAc confirmed increased uptake of the GalNAc-glycosylated peptide by the DCs (Figure 4E–F). The GalNAc MUC2 peptide predominantly co-localized with lysosomal marker LAMP-2, while non-glycosylated MUC2 fusion peptide predominantly co-localized with early endosomal marker EEA-1 (Figure 4E–F, Figure S1). In conclusion, GalNAc glycosylation increases uptake, but prevents processing of the two fusion peptides containing MHC class I binding epitopes.

## Discussion

Cancer associated aberrant glycans represent potent tumor antigens [11], [12]. Using MUC1 and ovalbumin as model molecules we present data suggesting that GalNAc glycosylation increases antigen uptake, MHC class II presentation, and CD4+ T cell activation inducing potent antibody responses. In contrast, GalNAc glycosylation may inhibit MHC class I antigen presentation and activation of antigen specific CD8+ T cells.

It is clear that GalNAc glycosylation potentiates antibody responses and T-cell proliferation after immunization with a GalNAc modified MUC1 peptide (degMUC1) (Figure 3A). An explanation for this GalNAc induced antibody response *in vivo* is likely to be the increase in antigen presentation, and following T-cell stimulation observed upon GalNAc glycosylation of the model peptide degMUC1 containing an OVA derived I-A^b^ binding peptide (Figure 1). This is in accordance with our previous finding of strong IgG responses to GalNAc MUC1 in human MUC1 transgenic mice [11], [12] and cancer patients [13], [14]. Furthermore, it is consistent with improved CD4+ T cell responses to OVA conjugated to GalNAc residues in other studies [59]. The ability of GalNAc to break immunological tolerance and to aid the generation of a CD4+ T cell dependent humoral immune response is of particular importance in cancer immunotherapy aimed at generating IgG antibodies. Moreover, the specific induction of CD4+ T cells has been suggested to lead to tumor eradication by delayed type hypersensitivity responses [64] and recently cancer patients have been cured after adoptive transfer of CD4+ T cells with tumor reactivity [65].

The immunodominant H2-K^b^ binding peptide SIINFEKL, derived from OVA, was used as readout for MHC class I presentation. Here, GalNAc glycosylation of the flanking MUC1 peptide inhibited MHC class I presentation of SIINFEKL on DCs as well as the activation of a specific CD8+ T cell response (Figure 2). In accordance with these findings, GalNAc glycosylation inhibited induction of CD8+ T cell responses *in vivo* after immunization of HLA-A2 transgenic mice with a GalNAc glycosylated degMUC1 peptide containing the HLA-A*02∶01 binding epitope (ALGSTAPPV) as compared to the non-glycosylated variant (Figure 3D–E). This could partly be explained by the diminished stability of the HLA-A*02∶01-ALGSTAPPV complex when the peptide is GalNAc-glycosylated. However, although HLA-A*02∶01 binding affinity and stability of the GalNAc-glycosylated peptide is slightly reduced it still appears to bind at a level that is sufficient for immunogenicity. Thus, our observations suggest alternative explanations. One possibility is that glycopeptides alter DC activation, thereby changing the T-cell activation. To support this it has been shown that selective MUC1 glycoforms induce T cell tolerance [66]. However, the inhibitory effect most likely takes place during processing since less peptide/MHC complexes reached the surface of the DCs. Thus, the effect of GalNAc on the CD8+ T cell response could be due to inhibition of proteasomal cleavage. This explanation has also been confirmed by biochemical studies of cleavage of MUC1 derived peptides by purified immunoproteosomal enzymes [60]. In this study, glycosylation at both the –VTS– and –GST– sites in the MUC1 tandem repeat sequence (VTSAPDTRPAPGSTAPPAHG) resulted in total block of antigen processing, while GalNAc glycosylation in only one of these sites inhibited antigen processing. This would explain our partial presentation of the CD8+ epitope, SIINFEKL, when only two GalNAcs were present in the SIINFEKL-MUC1 fusion peptide, while glycosylation with four GalNAcs per repeat lead to an almost total block of presentation. In agreement with our *in vitro* findings, it has been demonstrated that CD8+ T cell function is inversely correlated with the extent of glycosylation of the MUC1 protein used for priming of the CD8+ T cells [67]. This supports the notion that glycosylation may represent a problem in CD8+ T cell induction.

Increased antigen uptake by APCs is of great importance in determining T cell responses [19], [20]. It has been demonstrated that GalNAc MUC1 coated onto fluorescent microbeads are internalized by human DCs through the binding to macrophage galactose-type C-type lectin (MGL) and delivered to HLA class 1 and 2 compartments within the DC [19], [20]. It has also been shown that GalNAc conjugated OVA protein targets MGL and provides a better uptake and CD8+ T cell response [59]. This is in contrast to our findings with small peptide antigens. The glycosylation density might in part explain this. From our results it is clear that densely glycosylated 60 mer MUC1 in complex with streptavidin and coated onto microbeads induced markedly increased uptake (Figure 4A). In order to test if introduction of multiple GalNAc residues increased CD8+ T cell activation, we created a densely glycosylated MUC2 fusion peptide with a cleavable linker between the two peptide elements [68], [69]. MUC2 was chosen because it has multiple glycosylation sites with much higher density than MUC1 resulting in a peptide with over twice as many GalNAc residues on the 25 amino acid sequence. As expected, GalNAc MUC2 fusion was taken up more efficiently than the non-glycosylated counterpart (Figure 4B). However, this was not reflected in the activation of specific CD8+ T cells or peptide/MHC surface expression (Figure 4C–D). Presumably, the dense GalNAc glycosylation blocks CD8+ T cell activation, either by inhibiting antigen processing or by directing the immunogen to non-MHC I compartments. Supporting the latter, confocal microscope imaging demonstrated co-localization of internalized GalNAc MUC2 fusion protein with LAMP-2. In contrast, non-glycosylated MUC2 co-localized with the early endosomal marker EAA-1 (Figure 4E–F, Figure S1). It should be noted, however, that a robust induction of GalNAc-MUC1 and MUC1 specific CTLs have been reported in murine studies using a TLR-2 agonist adjuvant linked to a polio derived T helper epitope and a single GalNAc glycosylated tandem repeat MUC1 [70]. The degree and localization of glycans, inclusion of helper epitopes, and the route of uptake are of high importance for the immune response.

In conclusion, our data suggests that aberrant GalNAc O-glycosylation may inhibit the generation of a cancer specific CD8+ T cell response despite increased antigen uptake by DCs. This could be a potential pitfall for design of MUC1 targeting cancer vaccines. The creation of fusion peptides with two or more CD4+ and CD8+ T cell antigens, in which specific cleavage and processing sites have been introduced, might overcome this problem. However, such approaches might prove difficult because inhibition of CD8+ T cell responses has been observed even when the GalNAc residues were separated from the CD8+ T cell epitope with a linker sequence. In conclusion, GalNAc glycosylation of peptide antigens may boost the generation of CD4+ T cell responses, but inhibit CD8+ T cell responses.

## Materials and Methods

### Ethics Statement

All animal experiments were approved by the local animal facility and the Danish Veterinary and Food Administration with the approval number: 2008/561–1460. The animals were monitored daily after injection to detect any unwanted side effects and sacrificed by cervical dislocation at the end of the experiment.

### Peptides

MUC1 60 mer-peptide (VTSAPDTRPAPGSTAPPAHG*)*_3_ with and without biotinylation (Bio) was obtained as previously described [19], MUC2 (ITTTTTVTPTPTPTGTQTPTTTP), MUC2 fusion peptide (Biotin-PTTTPITTTTTVTPTPTPTGTQTPTAAAAAAPSIINFEKL), degenerated (deg) MUC1 24 mer (AHGVTSAPDNRPALGSTAPPVHNV), 9 meric peptide ALGSTAPPV and OVA-MUC1 fusion peptides (Table 1) were purchased from Schafer-N (Denmark) and glycosylated *in vitro* using recombinant human glycosyltransferases GalNAc-T2, -T4 and -T11 as previously described [12]. Concentrations of all peptides were equilibrated by HPLC standard curves and degradation of the peptides in serum was negligible for the relevant 48 h period (data not shown). KLH conjugated peptides were made as previously described [11].

### Measuring Peptide-MHC-I Affinity and Stability

The peptide-HLA-A*0201-affinity measurements were performed using a homogenous AlphaScreen based assay [71]. pMHC-I stability was measured using a homogenous scintillation proximity assay [72].

### Cell Lines

The following OVA peptide/MHC complex specific hybridomas were used: ISQAVHAAHAEINEAGR/I-A^b^ complex specific T cell hybridoma BO-97.10 [73] was a kind gift from John Kappler and Philippa Marrack and the SIINFEKL/H-2K^b^ complex specific B cell hybridoma 25- D1–16 [74] was a kind gift from Ronald D Germain (NIH). SIINFEKL/H-2Kb complex restricted T cell hybridoma RF33.70 [75] was also used. All cells were kept in RPMI 1640 with glutamax, 100 U/ml penicillin and 0,1 mg/ml streptomycin as well as 10% FCS (standard medium). Further 5% enrichment supplement were added T cell hybridomas. The enrichment supplement contains: glucose, essential and non-essential amino acids, ethanolamine, Na.pyrovat, insulin, testosterone, 2-ME and linoleic acid. For the B cell hybridoma 5% FCS, 0,1% of SSR-3 and 5% of enrichment supplement was added.

### Mice and Immunization Protocol

All mice were kept in the animal housing facilities of the Faculty of Health and Medical Sciences (University of Copenhagen). All experiments have been approved by the Danish authorities. Female Balb/c wild type (Taconic) and B6.Cg(CB)-Tg(HLA-A/H2-D)2Enge/J (HLA-A2 transgenic mice) (Jackson) were injected subcutaneously with 20 µg of (glyco-) peptide in a total volume of 200 µL (1∶1 with Freunds adjuvant (SSI, Denmark). The mice were immunized three times 10 or 14 days apart and blood samples were obtained by eye bleeding between 7 and 10 days after the last immunization. Lymphocytes were harvested from the spleen and lymph nodes. Wild type C57BL/6 mice (Taconic) were used for DC generation.

### ELISA

For the serum samples, ELISA plates were coated with 1 or 2 ug/mL of (glyco-) peptide, blocked with BSA incubated with sera (diluted 1∶100–1∶3200) and developed with HRP-conjugated goat anti-mouse IgG (Dako Denmark). TMB one-step substrate was used in detection (Dako, Denmark). Control sera from mice immunized with adjuvant only and naïve mice were included. mIL-2 ELISA was performed using the ELISA kit (BD Bioscience cat number: 555148 ) following the manufacture’s recommendations.

### T cell Proliferation

Lymphocytes were harvested from spleens and lymph nodes of immunized mice. Approximately 400,000 lymphocytes/well were stimulated with 100 µg/ml of peptide in 96-well round-bottom plates (Nunc, Roskilde, Denmark). The cells were cultured in standard medium with 0,1% 2-ME and 1,5% autologous serum. PPD and PBS were used as positive and negative controls. After 4 days of incubation the proliferation was assessed by a standard 20 hour [^3^H]-thymidin incorporation assay.

### CD8+ T cell Expansions

Spleen cells from HLA-A2 transgenic mice were re-stimulated *in vitro* with 10 µM ALGSTAPPV peptide, with or without glycosylation for 12 days (50 U/ml IL-2 was added at day two) followed by re-stimulation for an additional 10 days with peptide pulsed syngenic spleen cells. The presence of activated CD8+ T cells was assessed by intracellular flow cytometry for IFN-γ.

### Flow Cytometry

Intracellular flow cytometry was performed using the Cytofix/perm kit (BD Bioscience 554715) following the manufacture’s recommendations. Lymphocytes from spleen or lymph nodes were pulsed *in vitro* for 4 hrs with 60 µM ALGSTAPPV peptide +/− glycosylation and with Golgi stop. Anti-CD8a (FITC), anti-IFN-gamma (PE) and isotype controls all from BD Pharmingen.

Murine DCs were characterized in a standard surface staining flow cytometry assay by DC marker expression, using anti-CD80 (PE), CD86 (PE), MHC II (I-A/I-E) (PE) and CD11c (PE/FITC) antibodies and isotope controls, all from BD Pharmingen. The surface expression of SIINFEKL/H-2 K^b^ complexes was determined using 25-D1–16 B cell hybridoma supernatant and FITC labeled goat anti-mouse IgG1antibody from Southern Biotech. Secondary background staining was determined for all samples. All samples were run on a BD Facs Calibur and analyzed by Cell Quest Pro software.

### Murine DC Development, Purification and Co-culture

*In vitro* developed DCs were generated as described previously [76]. Cells were harvested at day six and resuspended in fresh medium supplied with the appropriate (glyco)peptides for 48 hours. FACS analysis showed at least 60% of the DCs was CD11c+ [76] (Figure S2). CD11c+ DCs were purified from C57BL/6 spleen cells using the MACS CD11c microbeads from Miltenyi Biotec and as per the manufacture’s recommendation. The DCs loaded with 120 µM (H-2K^b^ fusion), 3 µM –3 nM (I-A^b^ fusion) or 12-0.24 µM MUC2 fusion peptide was set up in co-culture with the appropriate T cell hybridoma. After 20 hrs the supernatant was harvested and stored at −80° until the IL-2 ELISA was performed.

### Uptake and Localization by Confocal Imaging

At day six the DCs were incubated with 60 µM Bio-MUC1 60 mer peptide +/− GalNAc for 4 hrs at 37°C. The surface was stripped using a glycine buffer and uptake was measured by an intracellular flow cytometry assay using AF488 labelled streptavidin from Invitrogen. Alternatively Bio-MUC1 60 mer peptide +/− GalNAc was mixed with the AF488 labeled streptavidin for 5 min on ice before addition to the DCs. Streptavidin alone was used as a control. The MUC2 fusion peptide +/− GalNAc was loaded onto the DCs at 12 µM concentration and incubated for 48 hrs at 37°C and uptake detected using flow cytometry by the AF488 labeled streptavidin. Streptavidin stained cells with no prior peptide loading and unstained cells were used as controls. The Bio-60 mer peptide +/− GalNAc was also coated onto red fluorescent NeutrAvidin labeled microspheres (580/605 nm, 1.0 Am; Molecular Probes) as previously described [19]. Localization studies were performed by confocal microscopy on a Zeiss LSM 710 confocal microscope. *In vitro* developed DCs were pulsed with 12 µM MUC2 fusion peptide +/− GalNAc for 2 hrs at 37°C, followed by staining with the AF488 streptavidin, anti-EEA-1, and anti-LAMP-2.

### Statistical Calculation

The unpaired two-tailed t test was applied to calculate statistically significance differences between the GalNAc deg MUC1 and the non glycosylated degMUC1 immunized mice.

## Supporting Information

Figure S1 **Co-localization MUC2 fusion peptide with endosomal/lysosomal markers.** Native or GalNAc modified MUC2 fusion peptide was allowed to internalize for 2 hr. After fixation and permeabilisation, cells were stained for LAMP-2 or EEA-1. Co-localization graphs of the intracellular co-localization of GalNAc (A) or non-glycosylated (B) MUC2 fusion peptide with early endosomal marker (EEA-1) or lysosomal marker (LAMP-2). X-axis depicts the AF488 labeled peptide and the Y-axis the AF594 (LAMP-2) or A546 (EEA-1) labeled organelle marker.
(PDF)

Figure S2 **DC development profile at day 6.** A) Gating of DCs. B-C) Flow cytometry staining for DC marker CD11c+ (B) and maturation markers CD80/86/MHCII (C) (green) and isotype control (purple). The positive cell population in the M2 gate constitutes ∼60% of total cell number.
(PDF)
