# Supplementary figures and images for: Cancer Associated Aberrant Protein O-Glycosylation Can Modify Antigen Processing and Immune Response
Source: PLoS One. 2012 Nov 26;7(11):e50139. doi: 10.1371/journal.pone.0050139 (PMC3506546; doi:10.1371/journal.pone.0050139)

Figure S1, Madsen *et al.*

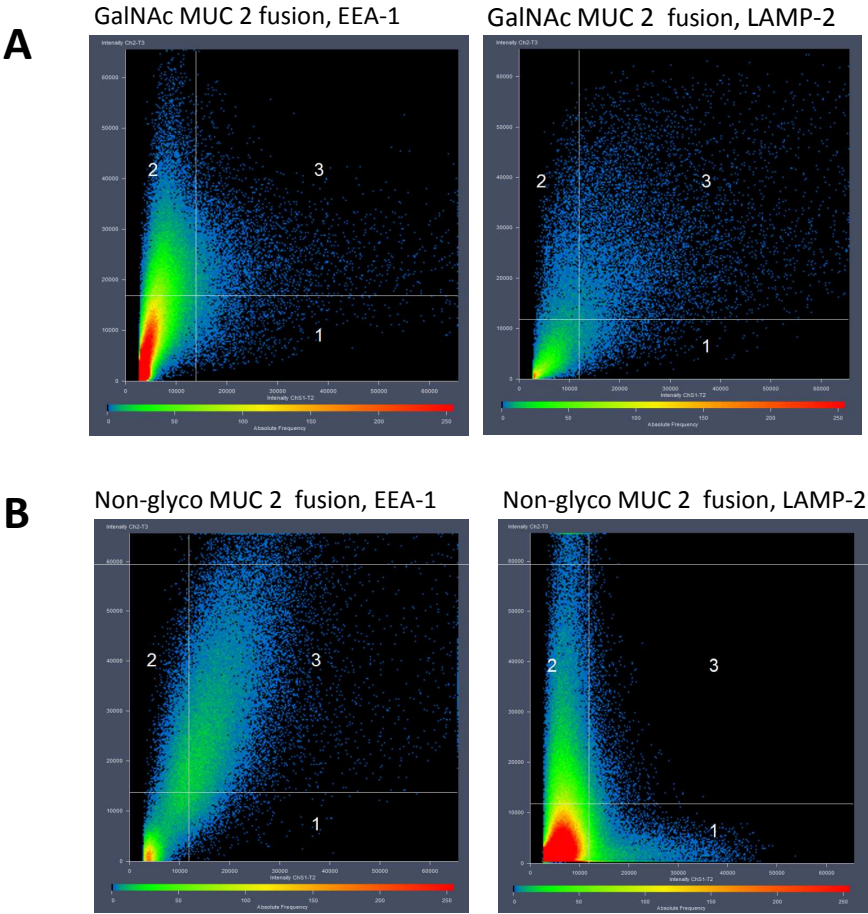

Supplement: Figure S1 — Co-localization MUC2 fusion peptide with endosomal/lysosomal markers. Native or GalNAc modified MUC2 fusion peptide was allowed to internalize for 2 hr. After fixation and permeabilisation, cells were stained for LAMP-2 or EEA-1. Co-localization graphs of the intracellular co-localization of GalNAc (A) or non-glycosylated (B) MUC2 fusion peptide with early endosomal marker (EEA-1) or lysosomal marker (LAMP-2). X-axis depicts the AF488 labeled peptide and the Y-axis the AF594 (LAMP-2) or A546 (EEA-1) labeled organelle marker. (PDF) [file pone.0050139.s001.pdf]

Figure S2, Madsen *et al.*

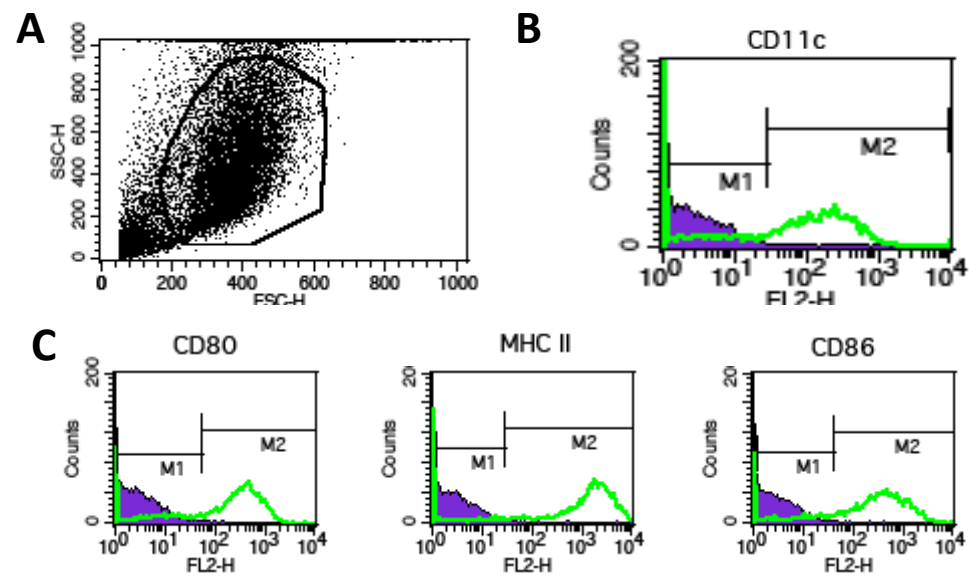

Supplement: Figure S2 — DC development profile at day 6. A) Gating of DCs. B-C) Flow cytometry staining for DC marker CD11c+ (B) and maturation markers CD80/86/MHCII (C) (green) and isotype control (purple). The positive cell population in the M2 gate constitutes ∼60% of total cell number. (PDF) [file pone.0050139.s002.pdf]
